# The Forty-Eight-Hour Week

**Published:** 1919-11-22

**Authors:** 


### THE FORTY-EIGHT-HOUR WEEK.

The pursuit of the general adoption of a forty-eight-hour working week in all hospitals, institutions, and wherever nurses are employed is making appreciable headway. It is, however, causing severe jolts in the comfortable ruts of tradition, and disturbing the peace of mind of various committees and Boards of Guardians. It is evident that the circulation of the Report and the inquiry into the general conditions of employment of nurses, instituted by the Council of the College of Nursing, has stirred the apathy existent for many years into an inquiring activity. In some instances there is indignant opposition; in others a rapid acquiescence, suggestive of a need to apply a quick remedy to cover an already acknowledged evil; and by others there is the grasping of an opportunity to right an obvious wrong. The adoption of a working week of forty-eight hours is under consideration throughout the Poor-Law Service in the Metropolis. Several London hospitals have the scheme already in working order, and the general adoption of the principle by all large and small institutions in London and the provinces is confidently hoped for in the near future.

The many discussions for and against a forty-eight-hour week afford some insight into the minds of those who have the control of nurses' lives in institutions. At Dewsbury the proposition to adopt the forty-eight-hour working week was carried finally by the strong support of several Labour members. The opposition to the measure by one member of that Board was based on the ground that, apart from the increased expenditure of £2,000 a year that it would involve, it was an "unreasonable proposition for employees engaged upon such work as that performed by their staffs." At Lynn, one member refused to believe that the nurses worked eighty-four hours a week, although proof was forthcoming, and further contended that their work was not of a laborious character. Other managers are more temperate and guarded, making extensive inquiry and effecting preliminary concessions before finally adopting the new measures. The Lincoln Guardians have a long road of reform to travel before reaching the forty-eight-hour goal. Although they have considerably reduced their existing scale of working hours, the day probationer's duty hours still average 56¾ a week and the night working hours 67 a week. At a meeting of the Colchester Guardians the resolution was lost, five voting in favour and seven against.

From the reports and comments collected from various institutions scattered up and down the country it may be seen to what extent the conditions under which nurses work are dependent on persons outside the profession. The only remedy for over-long working hours lies in maintaining an adequate staff sufficient to fill gaps caused by illness or other causes. Usually all turns on the question of accommodation. This difficulty in many instances seems to be insurmountable, and much imagination and energy are required if the general principle of an eight-hour day is to be established throughout the profession.

